# Replication Study in Chinese Population and Meta-Analysis Supports Association of the 5p15.33 Locus with Lung Cancer

**DOI:** 10.1371/journal.pone.0062485

**Published:** 2013-04-30

**Authors:** Juntao Ke, Rong Zhong, Ti Zhang, Lifeng Liu, Rui Rui, Na Shen, Yu Sun, Li Liu, Liming Cheng, Xiao-Ping Miao

**Affiliations:** 1 State Key Laboratory of Environment Health (Incubation), Ministry of Education Key Laboratory of Environment & Health, Ministry of Environmental Protection Key Laboratory of Environment and Health, Wuhan, Department of Epidemiology and Biostatistics, School of Public Health, Tongji Medical College, Huazhong University of Science and Technology, Wuhan, China; 2 Department of Epidemiology and Biostatistics, School of Public Health, Guangdong Pharmaceutical University, Guangzhou, China; 3 Department of Laboratory Medicine, Tongji Hospital, Tongji Medical College, Huazhong University of Science and Technology, Wuhan, China; The University of Texas M. D. Anderson Cancer Center, United States of America

## Abstract

**Background:**

Common genetic polymorphisms on chromosome 5p15.33, including rs401681 in cleft lip and palate transmembrane 1-like gene (*CLPTM1L*), have been implicated in susceptibility to lung cancer through genome-wide association studies (GWAS); however, subsequent replication studies yielded controversial results.

**Methodology and Findings:**

A hospital-based case-control study in a Chinese population was conducted to replicate the association, and then a meta-analysis combining our non-overlapping new data and previously published data was performed to clearly discern the real effect of lung cancer susceptibility. In our study with 611 cases and 1062 controls, the minor allele T carrier (TT plus CT) group conferred an OR of 0.801 (95% CI = 0.654–0.981) under the dominant model. The meta-analysis comprising 9111 cases and 11424 controls further confirmed the significant association in the dominant model (OR = 0.842, 95% CI = 0.795–0.891). By stratified analysis, we revealed that ethnicity and study design might constitute the source of between-study heterogeneity. Besides, the sensitivity and cumulative analyses indicated the high stability of the results.

**Conclusion:**

The results from our case-control study and meta-analysis provide convincing evidence that rs401681 is significantly associated with lung cancer risk.

## Introduction

Lung cancer is the most common cancer throughout the world. Globally, it accounts for 13% (1.6 million) of the total cases and 18% (1.4 million) of the deaths in 2008 [1]. In China, the incidence and mortality rates of lung cancer have been increasing rapidly in the recent decades [2], and lung cancer is now the number one cause of cancer mortality [3]. Environment factors such as smoking and pollution have been established to increase the risk of lung cancer. However, the etiology is still unclear, and genetic factors are strongly suggested to play an important role in lung carcinogenesis, which was estimated to contribute 26 percent to the risk through twin study [4].

Recent genome-wide association studies (GWAS) have implicated multiple novel single nucleotide polymorphisms (SNPs) to lung cancer susceptibility [5]–[12]. rs401681, located in the intron 13 of *CLPTM1L* (cleft lip and palate transmembrane 1-like gene) [13], is one of the most studied SNPs. It was firstly identified in a GWA set of 1952 cases and 1438 controls, supported by pooling data with two other GWA studies (5095 cases and 5200 controls) and with replication in additional 2484 cases and 3036 controls [5], which were all within Caucasian population. And in Asians, Kohno et al. and Miki et al. both confirmed the association between the T allele of rs401681 and decreased risk of lung adenocarcinoma [14], [15]. However, the reported genetic effects varied across the following replication studies. For example, a study in Norway discerned that the rs401681 T allele wasn’t associated with the risk of non-small cell lung cancer (NSCLC) in 341 cases and 431 controls of Caucasians (*P*_trend_ = 0.259) [16], but another study with 1681 lung cancer cases and 1235 controls showed that the T allele was significantly associated with a reduced risk of overall lung cancer in the same ethnicity (*P* = 1.1×10^−5^) [17]. On the other hand, within Asians, Yoon et al. replicated the association between the SNP and NSCLC (*P*_trend_ = 1.89×10^−4^), thereafter, neither Bae et al. [18] nor Chen et al. [19] replicated the similar positive results under the allelic model.

As above, the outcomes remain ambiguous and conflicting, which is probably due to the modest effect of this SNP, leading to the lack of power in small genetic association studies. It is also potentially owning to the so-called “winner’s curse” phenomenon that OR of disease variant is usually overestimated in the first positive study. According to the reported OR of this study, the necessary sample size of replication studies would be underestimated, then the underpowered replication would be difficult to succeed [20]. Nevertheless, meta-analysis is an effective method combing data together to increase the sample size, getting enough power to clarify inconsistent results in genetic association studies [21]. A comprehensive meta-analysis of publications studying the associations between *TERT* locus polymorphisms and risk of different cancers in a time span of 2003–2011, has presented a modest risk reduction (per-allele OR = 0.87, 95% CI = 0.84 to 0.89) of rs401681 for lung cancer [22]. Two recently published meta-analyses further supported the similar findings under the allelic model [23] and additive model [24]. By contrast, we conducted a meta-analysis of four specific genetic models, which could provide more implications for the possible manners of inheritance of the SNP. Additionally, our study combined results from published studies up to 2012, including a set of unpublished data of genotype frequency requested from the original authors of a large-sample-size GWAS [12]. At the same time, we included the data of our own replication study within a Chinese population in the whole meta-analysis followed by stratified, sensitivity and cumulative analysis, providing a comprehensive and precise estimation of the association between rs401681 and lung cancer risk.

## Materials and Methods

### Study Population

In the present study, a total of 611 lung cancer cases and 1062 cancer-free controls were recruited from Tongji Hospital of Huazhong University of Science and Technology (HUST) between 2009 and 2011. All subjects were unrelated ethnic Han Chinese in Wuhan region. Cases were histopathologically confirmed with all lung cancer types and have not received any treatment prior to blood samples collection. Controls were selected randomly from a physical examination programs at the same hospital in the same time period as the patients were enrolled. The case patients and control subjects are adequately matched in terms of gender and age (±5 years). At recruitment, 5-ml peripheral venous blood was collected from each subject after informed consent was obtained. This study was approved by ethnics committee of Tongji Hospital of Huazhong University of Science and Technology.

### Genotyping

Genomic DNA was extracted from the peripheral blood sample applying the RelaxGene Blood System DP319-02 (Tiangen, Beijing, China) in accordance with the manufacturer’s instructions. The rs401681 was genotyped with the TaqMan SNP Genotyping Assay (Applied Biosystems, Fostercity, CA) on a 7900HT Fast Real-Time PCR System (Applied Biosystems, Fostercity, CA). 5% duplicated samples were randomly selected to assess the reproducibility for quality control, with a concordance rate of 100%.

### Statistical Analysis

The *χ^2^* test and *t* test were applied to estimated differences in demographic variables and distributions of genotypes between cases and controls. Hardy-Weinberg equilibrium (HWE) was calculated using goodness-of-fit *χ^2^* test for genotypes in controls and a value of *P*<0.05 was considered as significant disequilibrium. After adjusting for age, sex and smoking status, unconditional multivariate logistic regression was employed to estimate genotypic odds ratio (OR) and its 95% confidence interval (95% CI), with the reference of the common homozygote. In order to avoid the assumption of genetic models, dominant and additive models were also analyzed. All above statistical analyses were performed with the SPSS 20.0 software.

### Meta-analysis of rs401681 in Association with Lung Cancer Risk

A systematic literature searching on PubMed, EMBASE, and ISI Web of Science databases up to April 2012 was performed, using the keywords ‘*rs401681*, *CLPTM1L*, *TERT*, or *5p15.33′* combined with ‘*lung cancer*, *NSCLC’* without language restrictions. Basing on the above searching strategy, we further searched in Chinese Biomedical (CBM) database. Meanwhile, references listed in the retrieved articles were scanned. Reviews and comments were also checked for additional studies. Articles which met the following criteria were included: (1) case-control or nested case-control study assessing the association between rs401681 and lung cancer risk; (2) providing data for calculating genotypic odds ratio (ORs) with corresponding 95% confidence interval (95% CI); (3) genotypes in controls being in Hardy-Weinberg equilibrium (*P*>0.05) (4) studies of humans. Whenever studies pertained to overlapping subjects, the one with larger sample size was selected to avoid duplication.

The following information from each study was extracted: first author’s last name, year of publication, geographic location, ethnicity of study population, study design, genotyping method, numbers of cases and controls, frequencies of genotypes in cases and controls. Pooled frequency of the T allele in different ethnicities and histological types was estimated by the inverse variance method previously used by Thakkinstian et al [25]. The metrics for effect size of genotypes CT versus CC and TT versus CC were calculated, and a dominant model and an additive “per-allele” model were also considered. Here we used *χ^2^-*based Cochran’s *Q* statistic and the *I^2^* metric to assess between-study heterogeneity. Heterogeneity was considered significant at *P*<0.10. The subsequent cut-off points of the quantity *I^2^* were utilized to quantify heterogeneity: *I^2^* = 0−30%, no or marginal between-study heterogeneity; *I^2^ = *30%–75%, mild heterogeneity; *I^2^* = 75−100%, notable heterogeneity [26]. When homogeneity existed basing on *P* for *Q* statistic greater than 0.1, the fixed-effects model (Mantel-Haenszel method) was adopted to compute the pooled ORs and 95% CIs [27]; otherwise, the random-effects model (DerSimonian and Laird method) was applied [28]. Then we conducted stratified analysis, if feasible (the number of studies included in each subgroup is not less than 3), according to ethnicity (Asian and Caucasian), study design (GWAS and replication studies), and histological type (NSCLC and integrated lung cancer). A sensitivity analysis was also carried out to assess the influence of each study on overall pooled OR, with sequential omission of individual study [29]. To investigate the dynamic trend of the association, cumulative analysis was performed by assortment of publication times [30]. Finally, publication bias was estimated by Egger’s test [31]. All statistical analyses were implemented by Stata 11.0 software and all *P* values are two-tailed with a significant level at 0.05 except for *Q* test for heterogeneity.

### Bioinformatics Analyses

To predict the function of rs401681, bioinformatics analyses were conducted with three online bioinformatics tools“F-SNP” (http://compbio.cs.queensu.ca/F-SNP/), “FastSNP” (http://fastsnp.ibms.sinica.edu.tw/pages/input_CandidateGeneSearch.jsp) and “SNP Info” (http://snpinfo.niehs.nih.gov/snpinfo/snpfunc.htm).

## Results

### Results of Case-control Study

#### Population characteristics

A total of 611 incident cases of overall lung cancer and 1062 frequency-matched controls were enrolled in this study. As shown in Table 1, no statistically significant differences were found between cases and controls in terms of sex (*P* = 0.475) and age (*P* = 0.287) distribution. Males were 68.6% among cases compared with 70.2% among controls and the median age was 61.5 for cases and 61.0 for controls. As expected, more smokers were presented in the cases than in the controls (53.1% versus 43.1%; *P*<0.001), considering that cigarette smoking is the major etiological factor for lung cancer. Herein, smokers were defined as those who had smoked at least one cigarette per day for 12 months or longer at any time in their life, while non-smokers were defined as those who had not. Of the cases, 427 (69.9%) were histopathologically confirmed as NSCLC, the most common type that accounting for approximately 85% of lung cancer in general, including adenocarcinomas, squamous cell carcinomas and large cell lung carcinomas.

**Table 1:** The characteristics of the study population.

	Case (N = 611)	Control (n = 1062)	
Variables	NO. (%)	NO. (%)	*P*
Gender			0.475^a^
Male	419(68.6)	746(70.2)	
Female	192(31.4)	316(29.8)	
Median age	61.5	61.0	0.115^b^
≤61.0	322(52.7)	531(50.0)	0.287^a^
>61.0	289(47.3)	531(50.0)	
Smoking status			<0.001^a^
Never-smoker	279(45.7)	599(56.4)	
Smoker	324(53.0)	458(43.1)	
Unknown	8(1.3)	5(0.5)	
Histological type			
NSCLC	427(69.9)		
Others	184(30.1)		

a *P* value was calculated by the *x* ^2^ test;

b *P* value was calculated by the *t* test.

Abbreviations: NSCLC, non-small cell lung cancer.

#### Association analysis

The distribution of rs401681 genotypes among subjects are displayed in Table 2, and no significant difference was observed between cases and controls in the overall test (*P* = 0.073). Genotypes in controls complied with Hardy-Weinberg equilibrium (*P* = 0.932). In the multivariate logistic regression model adjusted for age, sex and smoking status, individuals with the CT genotype had a significant decreased risk of lung cancer (OR = 0.794; 95% CI, 0.640–0.984) compared to those with the CC homozygote. A dominant model was performed to increase statistical power, by combining the TT with the CT into a T carrier (TT plus CT) group. And the result showed that the T carriers also present significantly reduced risk (OR = 0.801; 95%CI, 0.654–0.981), suggesting a dominant effect of this polymorphism on cancer in Chinese population. Likewise, significant associations were found in the additive models, with per-T-allele OR of 0.856 (95%CI = 0.734–0.998). To explore the variant’s effect on NSCLC, we also compared the genotype distribution between the NSCLC cases and controls. Similar positive association and stronger genetic effect were found in this subtype of overall lung cancer (heterozygous model: OR = 0.736, 95%CI = 0.577–0.939; dominant model: OR = 0.740, 95%CI = 0.588–0.931; additive model: OR = 0.803, 95%CI = 0.674–0.958).

**Table 2:** Association between rs401681 and lung cancer risk in a Chinese population.

Genotype	Controls (n = 1062)N(%)	Cases (n = 611)N(%)	*P* †	Crude OR (95%CI)	*p*	Adjusted OR (95%CI)‡	*p* ‡
Successful genotype	1060(99.8)	602(98.5)					
*Overall*							
CC	493(46.5)	315(52.3)	0.073	1.000		1.000	
CT	459(43.3)	231(38.4)		**0.788(0.637, 0.974)**	**0.027**	**0.794(0.640, 0.984)**	**0.035**
TT	108(10.2)	56(9.3)		0.812(0.571, 1.154)	0.245	0.833(0.583, 1.191)	0.316
Dominant model				**0.792(0.648, 0.968)**	**0.023**	**0.801(0.654, 0.981)**	**0.032**
Additive model				**0.856(0.734, 0.998)**	**0.047**		
*NSCLC*							
CC	493(46.5)	229(54.4)	0.023	1.000		1.000	
CT	459(43.3)	155(36.8)		**0.727(0.572–0.924)**	**0.009**	**0.736(0.577–0.939)**	**0.013**
TT	108(10.2)	37(8.8)		0.738(0.492–1.106)	0.141	0.758(0.503–1.142)	0.185
Dominant model				**0.729(0.581–0.914)**	**0.006**	**0.740(0.588–0.931)**	**0.010**
Additive model				**0.803(0.674–0.958)**	**0.015**		

Abbreviations: OR, Odds ratio; 95%CI, 95% confidence interval.

† P values were calculated by the Pearson Chi-Square test.

‡ Data were calculated by logistic regression model after adjusting for age, sex, and smoking status.

However, when we further examined with stratifications by smoking status (smokers and non-smokers) and the median age (age≤61.0 and age>61.0), no valid associations were detected under the dominant model except in the group who were under the age of 61.0. Null association might be due to the small number of subjects after stratification in the current study. The significant finding of the younger group is in line with the conception that genetic susceptibility is often associated with an early age of disease onset. But when we particularly defined the early-onset cases as who were ≤50.0 years of age and performed stratification analysis, we observed no significant associations (Table S1).

### Results of Meta-analysis

#### Study characteristics

As shown in Figure S1, 13 eligible original publications including 15 studies [5], [7], [9]–[12], [14]–[16], [18], [19], [32], [33] were firstly identified and screened for retrieval, of which, 9 studies were judged to preliminarily fit the inclusion criteria. However, after further examination, one of the studies “Wang-Texas 2008” of the publication reported by Wang Y et al. [5] was excluded since the cases overlapped with the sample of a previous study reported by Amos CI et al [7]. Besides, we removed the study “Wang-IARC 2008” in our pre-analysis of data that mostly contributed to the original notable heterogeneity, which might be due to its mixed study population from several different countries [5]. Finally, 7 publications plus current study comprising 8 case-control studies of 9111 cases and 11424 controls provided data for this meta-analysis [5], [7], [12], [16], [18], [19], [33]. The characteristics of the included studies were presented in Table 3.

**Table 3:** The characteristics of included studies.

First author	Year	Country	Ethnicity	Study type	Genotyping method	Histological type	Case/control
Amos	2008	USA	Caucasian	GWAS	Illumina	NSCLC	1153/1137
Wang-UK	2008	UK	Caucasian	GWAS	Illumina	Lung cancer	1950/1438
Zienoldding	2009	Norway	Caucasian	Replication	TaqMan	NSCLC	341/107
Yoon	2010	Korea	Asian	Replication	Affymetrix&TaqMan&Invader	NSCLC	1425/3011
Bae	2011	Korea	Asian	Replication	LightCycler	Lung cancer	1086/1079
Hu	2011	China	Asian	GWAS	Affymetrix	Lung cancer	2326/3073
Chen	2012	China	Asian	Replication	TaqMan	Lung cancer	228/195
Current Study	2012	China	Asian	Replication	TaqMan	Lung cancer	602/1060

Abbreviations: GWAS, genome-wide association studies; NSCLC, non-small cell lung cancer.

#### Pooled frequency of risk allele

Under fixed model (either *P* for heterogeneity>0.10), pooled T allele frequencies were 44.6% (95CI = 43.4%–45.9%) in Caucasian controls and 32.0% (95%CI = 31.2%–32.7%) in Asian controls, which were similar to those reported in HapMap database of 43.4%, 31.0% and 35.8% for Caucasians, Chinese and Japanese respectively.

Significant between-study heterogeneity were observed in both subunits of NSCLC and lung cancer (*P* for heterogeneity<0.001). Under random-effects model, the pooled frequency of the T allele was 36.9% (95%CI = 26.2%–47.5%) in the NSCLC cases of our whole study, which was slightly higher than that of 31.6% (95%CI = 26.3%–36.8%) in overall lung cancer cases.

#### Overall meta-analyses of rs401681 in associated with lung cancer

**Figure 1:**
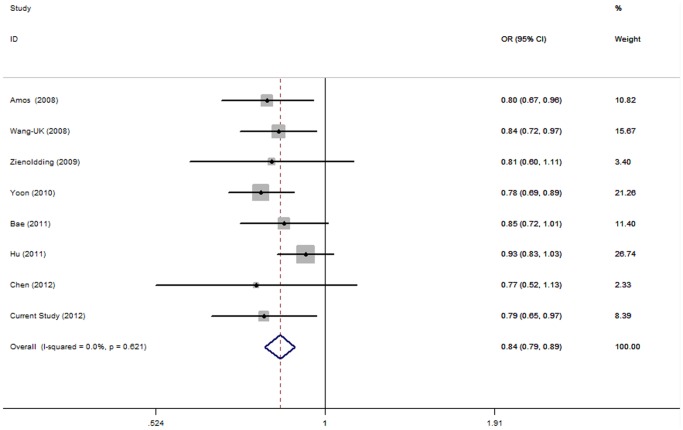
Forest plots of meta-analysis of association rs401681 with lung cancer under the dominant model.

No significant evidence of heterogeneity was seen in all genetic models except the homozygous model (*P* for heterogeneity<0.10), and its OR which was pooled under random-effects model are 0.778 (95%CI = 0.674–0.900). On the other hand, fixed-effects model was applied for heterozygous, dominant (Figure 1) and additive models without evident heterogeneity (each *P* for heterogeneity>0.10). All of these models conferred significant decreased risk of lung cancer, with ORs of 0.864 (95%CI = 0.813–0.917), 0.842 (95%CI = 0.795–0.891), 0.871 (95%CI = 0.835–0.908), respectively (Table 4). The meta-analysis of rs401681 revealed the similar results of significant decreased risk with lung cancer risk to our case-control study, on the same heterozygous, dominant and additive models.

**Table 4:** Meta-analysis of the rs401681 in association with lung cancer risk.

Category	Genetic model	OR(95%CI)	*P*	*I^2^* (%)	*P* forheterogeneity	*P* for Egger’s test
Overall (n = 8)	CT/CC	0.864(0.813–0.917)	1.94E-06	10.3	0.350	0.055
	TT/CC	0.778(0.674–0.900)	6.99E-04	51.3	0.045	0.088
	Dominant model	0.842(0.795–0.891)	3.64E-09	0.0	0.621	0.221
	Additive model	0.871(0.835–0.908)	1.25E-10	0.0	0.477	0.780
*Ethnicity*						
Caucasian (n = 3)	CT/CC	0.845(0.756–0.945)	3.18E-03	0.0	0.863	0.989
	TT/CC	0.759(0.657–0.877)	1.84E-04	0.0	0.900	0.318
	Dominant model	0.821(0.739–0.913)	2.52E-04	0.0	0.936	0.724
	Additive model	0.867(0.808–0.930)	7.62E-05	0.0	0.933	0.077
Asian (n = 5)	CT/CC	0.872(0.811–0.936)	1.70E-04	45.2	0.121	0.094
	TT/CC	0.825(0.637–1.070)	1.47E-01	71.8	0.007	0.111
	Dominant model	0.850(0.794–0.910)	3.16E-06	18.2	0.299	0.397
	Additive model	0.873(0.829–0.920)	3.86E-07	37.3	0.172	0.700
*Study design*						
GWAS (n = 3)	CT/CC	0.907(0.836–0.985)	2.03E-02	36.0	0.209	0.062
	TT/CC	0.764(0.678–0.862)	1.13E-05	0.0	0.992	0.225
	Dominant model	0.875(0.810–0.946)	7.79E-04	18.4	0.294	0.117
	Additive model	0.888(0.840–0.938)	2.57E-05	0.0	0.763	0.331
Replication (n = 5)	CT/CC	0.815(0.746–0.891)	6.80E-06	0.0	0.799	0.275
	TT/CC	0.831(0.608–1.136)	2.46E-01	72.2	0.006	0.134
	Dominant model	0.804(0.738–0.875)	3.98E-07	0.0	0.946	0.950
	Additive model	0.849(0.796–0.905)	6.56E-07	19.0	0.294	0.323
*Histological type*						
NSCLC(n = 3)	CT/CC	0.831(0.750–0.921)	3.85E-04	0.0	0.940	0.773
	TT/CC	0.665(0.568–0.780)	4.66E-07	31.1	0.234	0.929
	Dominant model	0.792(0.718–0.873)	2.73E-06	0.0	0.960	0.269
	Additive model	0.823(0.766–0.884)	9.21E-08	0.0	0.503	0.726
Lung cancer (n = 5)						
	CT/CC	0.882(0.819–0.950)	9.33E-04	41.5	0.145	0.831
	TT/CC	0.819(0.729–0.920)	7.52E-04	46.9	0.111	0.573
	Dominant model	0.869(0.810–0.933)	1.00E-04	0.0	0.569	0.803
	Additive model	0.898(0.852–0.946)	4.72E-05	0.0	0.837	0.639

#### Stratified analyses

To explore the source of heterogeneity, stratified analysis was performed (Table 4). After stratified by ethnicity, all genetic models presented significantly decreased risk of lung cancer and showed hardly any heterogeneity (*P* for heterogeneity>0.10, *I^2^* = 0) in Caucasian. In Asian population, decreased risk was also conferred without evidence of heterogeneity in all genetic models except for the TT genotypic model (pooled OR = 0.825, 95%CI = 0.637–1.070, *P* for heterogeneity<0.10, *I^2^* = 71.8%). The mildly reduced risk from one copy of T allele to two copies in Chinese (Table 2) or Asian population (Table 4), potentially pointed out that the T variant of rs401681 acted in different manners between different ethnical populations. According to study design, statistically significant findings were seen in the GWAS without heterogeneity, but negative outcomes appeared in the homozygous model again within the subgroup of replication studies. Regarding the histological type, significant association was observed in all four genetic models of our study in either NSCLC or integrated lung cancer. It is worth noting that larger effect of the minor allele T was seen in NSCLC than that in overall lung cancer, which could support the same finding of our case-control study.

#### Sensitivity analysis

To evaluate the affection of individual study on the combined estimate, a one-way sensitivity analysis was performed, by dropping each particular data set at a time. As shown in Table 5, a series of pooled OR with 95% CI were not materially altered before or after each elimination of study under the dominant model, indicating that our results were robust.

**Table 5:** Sensitivity analysis of dominant model.

Study omitted	OR (95%CI)	*P* forheterogeneity	*I^2^*
Amos 2008	0.847 (0.797–0.899)	0.545	0.0%
Wang-UK 2008	0.843 (0.792–0.897)	0.505	0.0%
Zienoldding 2009	0.843 (0.795–0.893)	0.509	0.0%
Yoon 2010	0.858 (0.804–0.914)	0.712	0.0%
Bae 2011	0.840 (0.791–0.893)	0.507	0.0%
Hu 2011	0.810 (0.757–0.867)	0.987	0.0%
Chen 2012	0.843 (0.796–0.894)	0.531	0.0%
Current Study 2012	0.846 (0.797–0.898)	0.552	0.0%

Since significant between-study heterogeneity for the Asian studies was observed in the TT genotypic model, we also performed a sensitivity analysis among those studies. And we have found that the heterogeneity reduced most (*I* ^2^ = 15.6%) when the study of Yoon et al. was excluded (*I* ^2^ = 56.2%, *P* = 0.077), with the same null association (OR = 0.909, 95%CI = 0.705–1.172).

#### Cumulative meta-analysis

**Figure 2:**
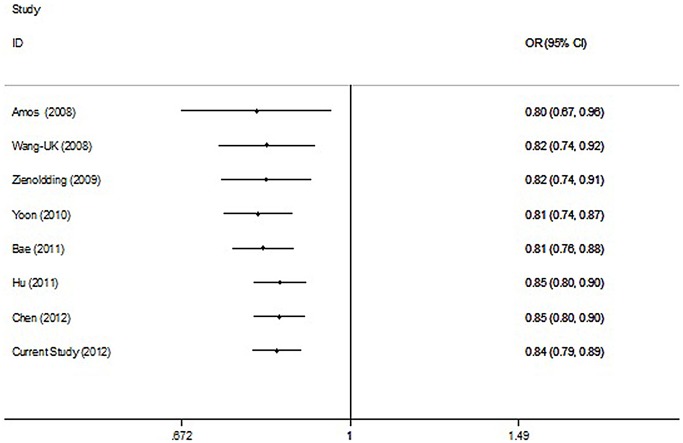
Forest plots of cumulative meta-analysis of association rs401681 with lung cancer under the dominant model.

Cumulative meta-analysis was carried out via assortment of studies in chronologic order. As shown in Figure 2, inclinations toward significant association were obvious over time in the dominant model. Simultaneously, the 95% CIs became increasingly narrower with accumulation of more data, suggesting the progressively improved precision of the estimates by continual enlarging sample size.

#### Publication bias

The results of Egger’s test indicated that there was no publication bias in all four genetic models (all *P* for Egger’s test>0.05, Table 4).

### The Bioinformatics Analyses of rs401681

Among three bioinformatics tools, two of them, “F-SNP” and “FastSNP” consistently forecasted that the SNP was likely to be functional as transcriptional regulation.

## Discussion

In this compound study, we replicated the significant association between rs401681 and lung cancer risk in the Chinese population. Also, the following meta-analysis integrating data from the current and 7 previously published studies suggested that the SNP was significantly associated with reduced lung cancer risk under genotypic, dominant and additive models. Sensitivity analysis indicated the stability of the result and cumulative analysis further confirmed the positive findings, showing the effect of the variant got progressively significant with accumulation of more data.

These findings are biological plausible to some degree. rs401681 is located at 5p15.33, a susceptibility region for lung cancer encompassing two known genes *TERT* (telomerase reverse transcriptase) and *CLPTM1L* (cleft lip and palate transmembrane protein 1-like). Although little was known about the function of rs401681 which was situated within the intronic region of *CLPTM1L*, our bioinformatics analysis indicated that it might be transcription regulatory and further affect the expression of the gene. *CLPTM1L*, named for its homology with Cleft Lip and Palate Transmembrane Protein 1 that disrupted in a family with cleft lip and palate [34], was identified as an up-regulated transcript in a cisplatin resistant ovarian tumor cell line [13]. Over-expression of *CLPTM1L* mRNA was discovered in many kinds of cancer [32], [35], [36]. The function of *CLPTM1L* and its role in tumorigenesis is thus far unknown. But a recent study reported that *CLPTM1L* was a commonly overexpressed anti-apoptotic factor in lung cancer, suggested it a inhibitor role in genotoxic stress induced apoptosis, and therefore identified *CLPTM1L* as an important factor influencing survival of DNA damaged tumor cells and potentially lung cancer susceptibility [37].

*CLPTM1L* might be relevant not only in the light of its own biological activity but also because it is in LD with *TERT*. The entire gene *CLPTM1L* resides in a 62-kb region of high linkage disequilibrium (LD) that encompasses the 5′-end of *TERT*, its promoter [38]. *TERT* is the reverse transcriptase component of telomerase [39], making it essential for production of telomerase enzyme which is responsible for telomere regeneration [40]. And regeneration of telomeres is highly suggested to be a vital step for carcinogenesis of most cancer [41]. The C allele of rs401681 was reported to be associated with the shortening of telomere length [32], which is in favor of its involvement in telomere biology and even cancer development. It is possible that the polymorphism of *CLPTM1L* is in linkage disequilibrium with some causal locus in the promoter of *TERT* which are hitherto uncharacterized.

The results of our case-control study indicated that the minor allele T of rs401681 was significantly associated with protective effect to lung cancer in heterozygous, dominant and additive genetic models, but not in the homozygous possibly because of our relative small sample size. Despite similar significant relationship maintained in our meta-analysis in all genetic models, obvious between-study heterogeneity can not be ignored in the homozygous model. A comprehensive stratified analysis was conducted to interrogate the potential source of heterogeneity. After stratified by ethnicity, all genetic models of the T variant allele were significantly associated with reduced cancer risk with disappeared heterogeneity in Caucasians, while in Asians, all genetic models conferred decreased risk without significant heterogeneity except the homozygous model. The apparent difference between the two homozygous ORs implied different allele frequencies between Asians and Caucasians, which is supported by our study of pooled T allele frequencies and HapMap data as shown above. In view of the differences in allele frequencies and possible distinction of linkage disequilibrium patterns among populations, distinct genetic mechanisms with different genetic effect sizes for TT genotype between two populations may occur. As to the heterogeneity in Asian studies, suggested by the subgroup sensitivity analysis (data not shown), it may largely attribute to the study of Yoon et al with various kinds of genotyping methods, which are Affymetrix, TaqMan and Invader. Regarding study design, obvious heterogeneity disappeared in GWAS but still showed in replication studies, which could be also explained by the diverse genotyping methods of the replications. Because NSCLC accounts for the great mass of overall lung cancer cases to almost 85% as mentioned above, we pooled these two types of studies to enlarge sample size without concern to the potential bias of strengthening or weakening the real effect of the variant to a great extent. There weren’t much change of the heterogeneity in the subgroup of NSCLC or lung cancer overall, hinting that histological type was not likely the source of heterogeneity. At the same time, a more pronounced risk reduction for NSCLC suggested that the rs401681 variant might have larger effect on NSCLC, supporting the same situation observed in case-control study. The variance of the genotypic effect between these two subgroups could be partly ascribed to different histological type characterized by allele frequency difference, with pooled T allele frequencies of 36.9% in NSCLC cases and of 31.6% in overall lung cancer. In the whole, ethnicity and study design probably accounted for the heterogeneity of the meta-analysis.

Despite the clear strength of the current comprehensive analysis strategy (a case-control study and a meta-analysis), several limitations should be pointed out. Firstly, the sample size of our case-control study was relatively small. Secondly, lung cancer is a complex trait related to environmental and genetic risk factors. However, insufficient environmental information limited us to further investigate the gene-environment interaction. Thirdly, lacking of functional experiments, whether this SNP is causal remained uncertain.

In conclusion, our replication study in a Chinese population and the subsequent meta-analysis collectively confirm genetic involvement of rs401681 polymorphism in lung cancer susceptibility, and suggested that the variant may yield stronger effect on NSCLC. However, fine-mapping of 5p15.33 region and functional experiments is warranted to identify causal variant.

## Supporting Information

Figure S1 **Follow chart of study selection.**
(TIF)

Table S1 **The association between rs401681 and risk of lung cancer by smoking status and age range.**
(DOC)

Checklist S1 **PRISMA checklist.**
(DOC)
