# Supplementary figures and images for: Replication Study in Chinese Population and Meta-Analysis Supports Association of the 5p15.33 Locus with Lung Cancer
Source: PLoS One. 2013 Apr 30;8(4):e62485. doi: 10.1371/journal.pone.0062485 (PMC3641186; doi:10.1371/journal.pone.0062485)

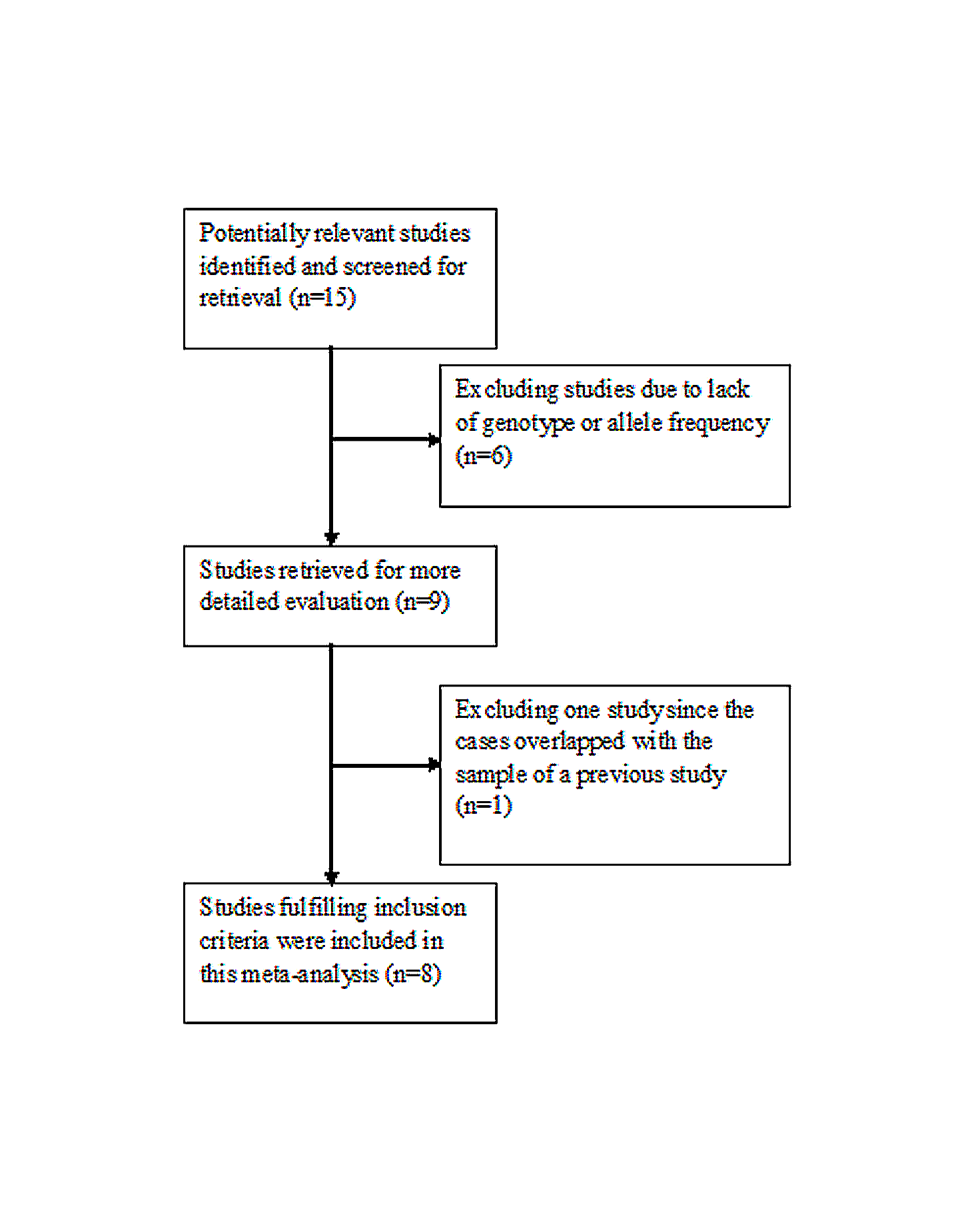

Supplement: Figure S1 — Follow chart of study selection. (TIF) [file pone.0062485.s001.tif]
