# Supplementary material for: Replication Study in Chinese Population and Meta-Analysis Supports Association of the 5p15.33 Locus with Lung Cancer
Source: PLoS One. 2013 Apr 30;8(4):e62485. doi: 10.1371/journal.pone.0062485 (PMC3641186; doi:10.1371/journal.pone.0062485)
Supplement: Table S1 — The association between rs401681 and risk of lung cancer by smoking status and age range. (DOC) [file pone.0062485.s002.doc]

**Table S1.** The association between rs401681 and risk of lung cancer by smoking status and age range.

| Subjects | Genotype | Control | Case | *P^†^* | OR (95%CI) | *p*^‡^ |
| --- | --- | --- | --- | --- | --- | --- |
| Smoking status |  |  |  |  |  |  |
| Smokers | CC | 214 | 168 | 0.150 | Reference | 0.145 |
|  | CT+TT | 242 | 154 |  | 0.808 (0.607-1.076) |  |
| Non-smokers | CC | 277 | 144 | 0.067 | Reference | 0.109 |
|  | CT+TT | 322 | 128 |  | 0.788 (0.589-1.055) |  |
| Median age |  |  |  |  |  |  |
| Age≤61.0 | CC | 233 | 178 | 0.001 | Reference | 0.002 |
|  | CT+TT | 296 | 141 |  | 0.638 (0.480-0.847) |  |
| Age>61.0 | CC | 260 | 137 | 0.880 | Reference | 0.916 |
|  | CT+TT | 271 | 146 |  | 1.016 (0.759-1.359) |  |
| Age range |  |  |  |  |  |  |
| Age≤50.0 | CC | 63 | 54 | 0.160 | Reference | 0.284 |
|  | CT+TT | 73 | 43 |  | 0.743 (0.432-1.279) |  |
| Age>50.0 | CC | 430 | 261 | 0.063 | Reference | 0.069 |
|  | CT+TT | 494 | 244 |  | 0.816 (0.655-1.016) |  |

^†^P values were calculated by the Pearson Chi-Square test.

^‡^Data were calculated by logistic regression model after adjusting for age, sex, and smoking status
